# Ribosome Assembly as Antimicrobial Target

**DOI:** 10.3390/antibiotics5020018

**Published:** 2016-05-27

**Authors:** Rainer Nikolay, Sabine Schmidt, Renate Schlömer, Elke Deuerling, Knud H. Nierhaus

**Affiliations:** 1 Institut für Medizinische Physik und Biophysik, Charité—Universitätsmedizin Berlin, 10117 Berlin, Germany; nierhaus@molgen.mpg.de; 2 Molecular Microbiology, University of Konstanz, Konstanz 78457, Germany; Sabine.Schmidt@uni-konstanz.de (S.S.); Renate.Schloemer@uni-konstanz.de (R.S.)

**Keywords:** protein synthesis as preferential target of antibiotics, ribosome as antibiotic target, inhibitors of ribosome assembly, concepts for identifying assembly inhibitors

## Abstract

Many antibiotics target the ribosome and interfere with its translation cycle. Since translation is the source of all cellular proteins including ribosomal proteins, protein synthesis and ribosome assembly are interdependent. As a consequence, the activity of translation inhibitors might indirectly cause defective ribosome assembly. Due to the difficulty in distinguishing between direct and indirect effects, and because assembly is probably a target in its own right, concepts are needed to identify small molecules that directly inhibit ribosome assembly. Here, we summarize the basic facts of ribosome targeting antibiotics. Furthermore, we present an *in vivo* screening strategy that focuses on ribosome assembly by a direct fluorescence based read-out that aims to identify and characterize small molecules acting as primary assembly inhibitors.

## 1. Introduction

Most antibiotics are microbial secondary metabolites mainly produced by actinomycetes. The production is usually induced by unfavorable growth conditions, when the producer has turned into the stationary or sporulation phase and its own metabolic activity is sharply reduced. Under these conditions, antibiotics will block the growth of better adapted competitors and thus prevent a further impairment of life conditions (for review, see [1]). 

Antibiotics therefore improve the survival chance of the producer, which represents the selective force for the natural development of antibiotics. Under these restricted conditions, synthesis of nucleic acids and protein synthesis is strongly reduced in the producer cell, which explains that, in many cases, the producer is not resistant against its own product.

**Figure 1:**
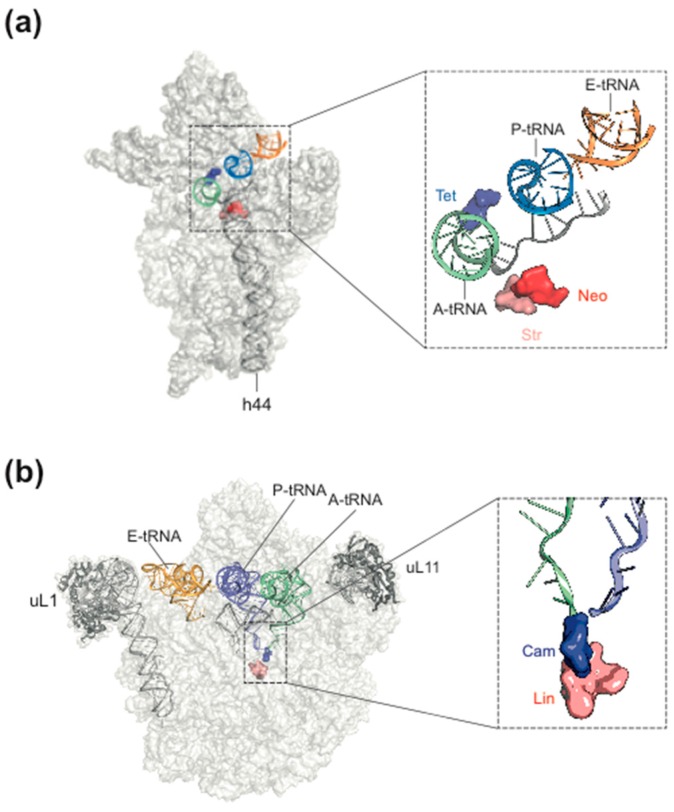
Antibiotic target sites of bacterial ribosomes. (**a**) a few antibiotic target sites on the 30S subunit mentioned in the text. Tet, tetracycline; Neo, neomycin, Str, streptomycin; (**b**) some antibiotic target sites on the 50S subunit. Cam, chloramphenicol; Lin, lincomycin. (from [7], modified, courtesy of Daniel N. Wilson, Munich Gene Center).

Antibiotics are chemically extremely diverse. The synthesis by microorganisms and a molecular weight less than 1000 Da are the only common features [2]. Nowadays, the term antimicrobials (short for antimicrobial substances) is used, which includes both natural products (antibiotics) and semi-synthetic or full-synthetic substances (chemotherapeutics). In addition to the wide chemical spectrum, the target spectrum is also enormous: More than 160 cellular targets have been described [3]. Examples are replication (e.g., nalidixic acid), transcription (e.g., rifamycin), translation (e.g., chloramphenicol) and the synthesis of the cell wall (e.g., penicillin). Most of the antibiotics inhibit the translating ribosome because, due to its complicated structure, it offers many interference points. The *Escherichia coli* ribosome contains 57 different components, 54 proteins and three ribosomal RNAs [4], and all of them are present in one copy per ribosome except the protein bL12, which is present in four copies. Nevertheless, the binding sites of the antibiotics are concentrated at and around functional hot spots of the small 30S and the large 50S subunit of the bacterial ribosome (Figure 1a,b). These sites are often dominated or exclusively built by elements of the rRNA (ribosomal RNA), and, importantly, the multiplicity of *rrn* operons present in most bacterial species aggravates development of resistance [5]. Mutations in only one gene have low impact and multiple mutations are of low probability [6].

Most of the natural antibiotics inhibit the peptidyltransferase center (PTC) on the 50S subunit (Figure 1a; [8,9,10]). Reasons for the prevalence of the PTC as antibiotic target are (i) the high number of crevices allowing binding of small molecules with high affinity; (ii) the fact that the PTC needs a high amount of structural flexibility and any interference by drug binding or mutations causing resistance might hamper the speed and/or accuracy of translation, which leads to the final outcome that any rescuing mutations within the PTC are associated with high fitness costs [10].

**Figure 2:**
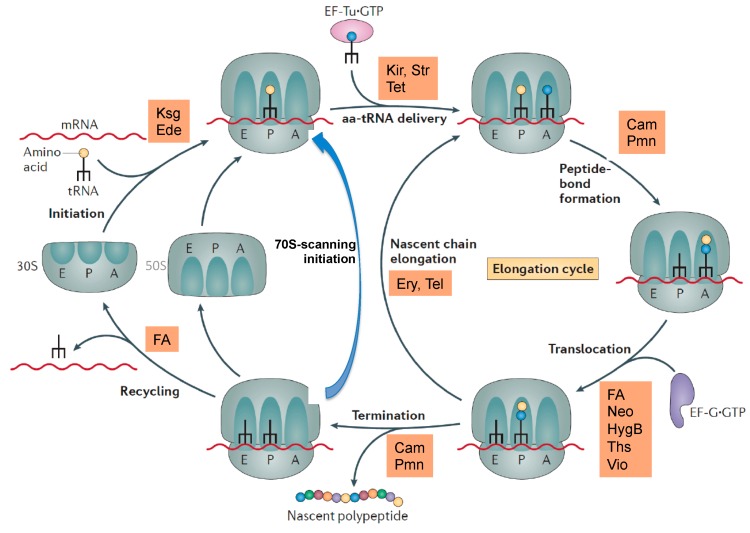
Overview of some antibiotics interfering with ribosomal functions. Kir, kirromycin; Str, streptomycin, tet, tetracycline; Cam, chloramphenicol; Pmn, puromycin; FA, fusidic acid; Neo, neomycin, HygB, hygromycin B; Ths, thiostrepton; Vio, viomycin; Ery, erythromycin, Tel, telithromycin. 70S-scanning initiation is a newly detected initiation mode in bacteria, which can replace the RRF/EF-G dependent recycling phase. Modified, from [11].

The most common target site of the 30S subunit is the decoding center of the A site, where the large group of aminoglycosides interferes with the fidelity of aminoacyl-tRNA(aa-tRNA)·EF-Tu·GTP selection (Figure 1b). Examples of binding sites on the 30S part of the A site outside the decoding center are the tetracyclines, which block stable binding of aa-tRNAs to the A site. The sketch of the ribosomal functions in Figure 2 summarizes the interference points of the various groups of antibiotics with ribosomal functions. Some antibiotics act directly on translational G-proteins. One example is kirromycin, which binds to the elongation factor EF-Tu·GTP and blocks its switch into the GDP conformer after delivery of the aa-tRNA to the A site. The result is that EF-Tu remains on the ribosome and thus blocks protein synthesis. Another example is fusidic acid (FA) acting on the second elongation factor G (EF-G). EF-G is responsible for the translocation of ribosomes to the next codon of the mRNA after a decoding step. FA also blocks the conformational switch into the GDP conformer of EF-G, which is necessary for the dissociation of this factor after it has fulfilled its ribosomal function.

Antibiotics are estimated to have originated between 2 billion. and 40 million years ago [12]. Consequently, resistance mechanisms have been evolving for the same large amount of time. Recently, 30,000-year-old bacterial isolates of Canadian permafrost soil confirmed that resistance predated the industrial use of antibiotics [12]. Five different types of resistance mechanisms are known:

(1) Mutations of membrane components affect the permeability barrier and, alternatively, transport proteins are affected, shifting the import:export ratio towards the export.
(2) The antibiotic target is altered by blocking binding or modifying the binding site, causing insensitivity to the drug. Mutation rates for types 1 and 2 are in the range of 10^−6^ to 10^−8^, *i.e.*, one bacterium out of 10^6^ to 10^8^ is resistant to the respective drug.
(3) Plasmids coding for enzymes that modify (acetylation, phosphorylation of adenylation) or degrade the antibiotic [3].
(4) Special factors remove the antibiotic from the target. For example, the EF-G derivative Tet(O) protein chases bound tetracycline off the ribosome [13,14]. Interestingly, resistance-types 1–4 are known for the tetracycline group [15].
(5) Rare mechanisms are dilution (overproduction) of the target molecule or activation of alternative pathways. Both are known for trimethoprim that inhibits dihydrofolate reductase [16].

Wasteful use of antibiotics in animal fattening and clinical applications have strengthened the spread of resistant and often multi-resistant germs via horizontal gene transfer [17], which is considered one of the major threads of medical therapies. It is therefore obvious that new drugs and possibly new targets are needed [5,18].

Facing the enormous accumulation of knowledge about all aspects of antibiotics, it is astonishing that the highly complicated process of assembly of the bacterial ribosomes does not seem to be a major target for antibiotics [19]. One possible reason is the lack or scarcity of suitable screening techniques to identify antibiotics specifically interfering with the ribosomal biogenesis. In this respect, some promising progress has been achieved in recent years, which we will consider in the following section.

## 2. Ribosome Assembly as Attractive Target for New Antimicrobials

**Figure 3:**
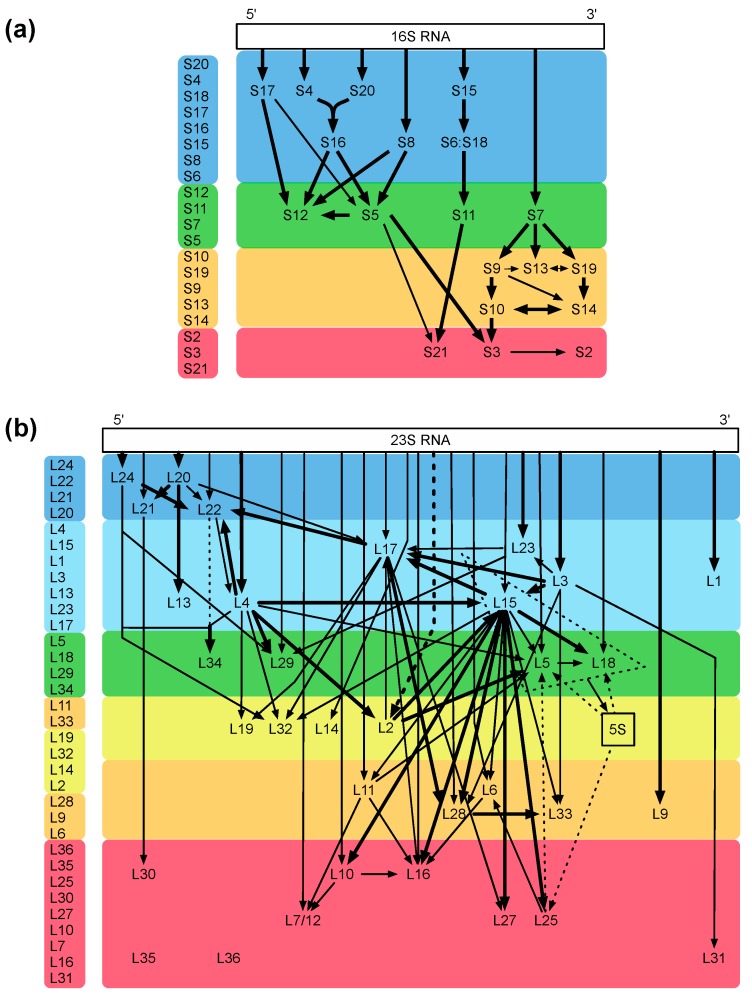
Assembly maps. (**a**) map of the small 30S subunit; (**b**) map of the large 50S subunit. The maps of Nomura (a) and Nierhaus (b) are color coded from blue to red according to their appearance during the assembly process *in vivo*, modified from Reference [28]. The assembly maps showing the assembly dependencies of the ribosomal proteins during the total reconstitution *in vitro* demonstrate an excellent agreement with the *in vivo* data. Please check if there is copyright issue.

The bacterial ribosome is one of the most intricate structures in the bacterial cell, and its assembly is a highly complex process. The fact that the small and large ribosomal subunits can be reconstituted *in vitro* [20,21] indicates that the complete information for the assembly process is intrinsically present in the sequences of ribosomal proteins and rRNAs [20]. With the help of the reconstitution technique, important mechanistic assembly steps could be unraveled, and examples are identification of the two assembly initiator proteins [22] and the five early assembly proteins necessary and sufficient for one of the most dramatic conformational changes known, which occurs during the assembly of precursor intermediates (a 33S particle becomes a highly compact 41S particle; [23]). Two ribosomal proteins, L24 and L20, could be mere assembly proteins without important functions in the mature ribosome [24]. The analyses cumulated in assembly maps for both the small and large subunit (Figure 3a,b; [25,26]; for review, see [27]).

What are the advantages of ribosome assembly as a target for antimicrobials? The inhibition of ribosome assembly precedes the interference with functions of the operating ribosome. Inhibition of early assembly events result in premature precursors, so called assembly dead ends, which, due to their loose structures, are cleared by proteases and RNases [29]. While such a scenario is irreversible, inhibition of translation is not. As soon as the inhibitor is gone, translation can recommence [30]. In addition, assembly differs significantly between bacterial and mitochondrial ribosomes [31]. This is an important notion because the latter ones are frequent targets of adverse reactions as has been shown for chloramphenicol [32], linezolid [33] and aminoglycosides [34].

## 3. Ribosome Assembly and Translation Are Coupled in Bacteria

**Figure 4:**
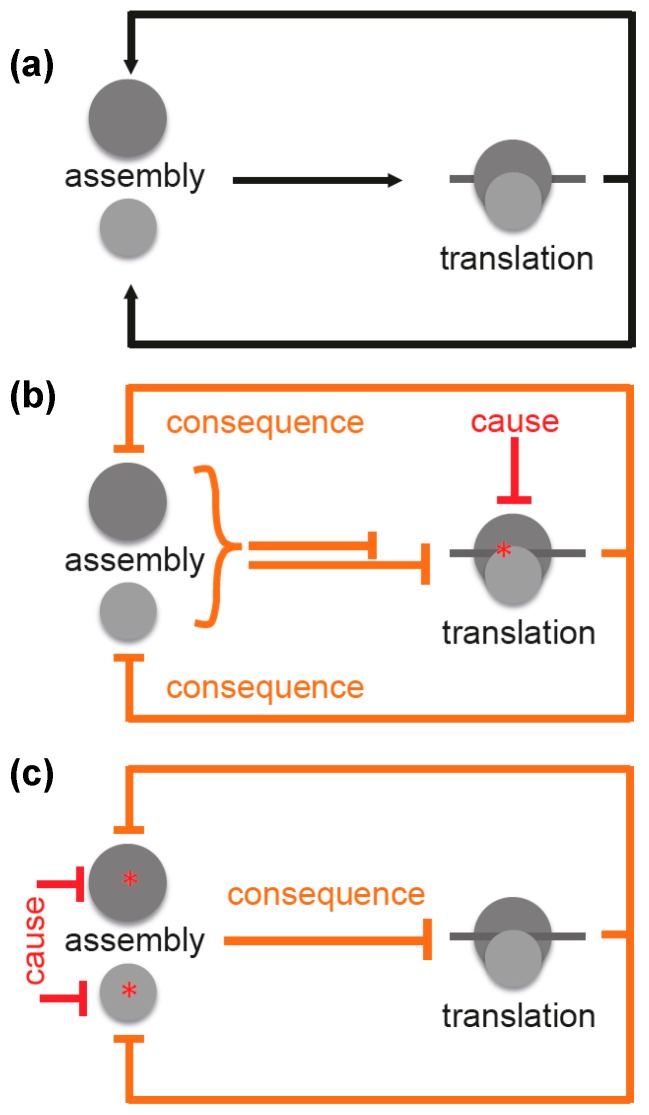
Scheme illustrating interconnectivity of ribosome assembly and translation. (**a**) ribosome assembly is the prerequisite for translation, which in turn is required for ribosome assembly; (**b**) inhibition of translation by a translation inhibitor is the causative mechanistic element. As a consequence, this results in inhibition (or at least reduction) of the assembly process, which, in turn, inhibits translation completing the negative feedback loop. Both processes are clearly interconnected *in vivo* and underlie complex regulation; (**c**) inhibition of ribosomal subunit assembly by a putative primary assembly inhibitor acting on one of the two subunits (cause) will result in inhibition of translation (consequence), which additionally inhibits assembly. This also creates a negative feedback loop. Legend: **Black** lines with arrows: positive effects; **red** lines: direct (causative) inhibitory effects; **orange** lines: resulting (consequent) inhibitory effects; asterisks: site of inhibition; **dark gray** circle: large ribosomal subunit; **light gray** circle: small ribosomal subunit; **dark gray** line: mRNA.

Facing both the highly complicated process of ribosome assembly and the fact that the majority of antibiotics inhibit functions of the bacterial ribosomes, it is surprising that hitherto no antibiotics have been detected, which primarily and specifically block ribosomal biogenesis. It is expected that translation inhibitors will affect also the synthesis of ribosomal proteins and thus the assembly process (Figure 4a). Consequently, assembly and translation are interdependent, which results in a chicken-and-egg problem [19].

For this reason, it is difficult to distinguish between cause and consequence. Inhibition of translation (cause) would result in a decrease in translation (consequence) (Figure 4b). However, this is difficult to distinguish from a scenario where inhibition of assembly would be the cause and a decreased translation the consequence (Figure 4c). This interdependence was observed by Nomura and coworkers, and they concluded that any inhibition of translation forces assembly defects due to the arising imbalance between rRNA and r-protein production [35]. 

Indeed, macrolides such as erythromycin have been demonstrated to block the assembly of the large 50S ribosomal subunit [36]. Chloramphenicol and erythromycin have been shown to decrease the production of some proteins (among others r-proteins). Precursor particles isolated upon antibiotic treatment were found to have reduced levels of exactly those proteins that were reduced in production [37,38]. Similarly, it has been described that the macrolide erythromycin and the ketolide telithromycin do not block the passage of nascent polypeptide chains through the exit tunnel completely but rather in a case-dependent manner. Biosynthesis of a number of polypeptides containing a newly identified *N*-terminal signal sequence did occur and was interpreted as a strategy to deregulate cellular pathways [39]. In that sense, ribosome assembly defects and metabolic dysregulation would be indirect effects caused by translation inhibitors.

Likewise, assembly of the small 30S subunit is inhibited by aminoglycosides such as streptomycin or neomycin [40]. However, it is likely that the primary target of the drugs are functions of the mature ribosome [37]. 

Taken together, it seems that identification of selective inhibitors of assembly requires the ability to distinguish between cause and consequence. A possible strategy to face that problem is to focus directly on subunit assembly.

## 4. Specific Readouts for Assembly Are Needed

**Figure 5:**
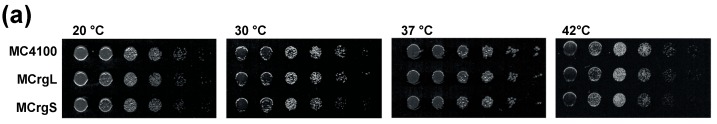

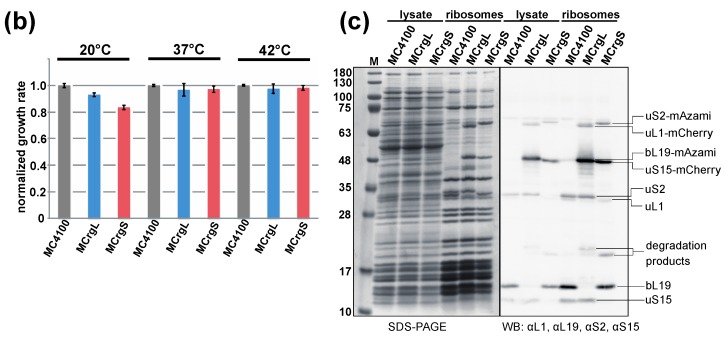
Phenotypic and biochemical characterization of strains MCrgL and MCrgS. (**a**) Serial dilutions of MC4100, MCrgL and MCrgS were spotted on LB-agar plates and incubated at temperatures as indicated; (**b**) LB cultures of the indicated cells were inoculated to a start OD_600_ of 0.05 and cultured at 20, 37 and 42 °C for 7 h in triplicates. Growth rates were calculated and normalized; (**c**) cell lysates and purified ribosomes obtained from the indicated strains were subjected to SDS-PAGE (**left** panel) or SDS-PAGE with subsequent immunoblotting (**right** panel). The SDS-PAGE was stained with Coomassie-brilliant blue and the Western blot membrane was probed with the indicated primary antibodies in combination with appropriated secondary antibodies. Proteins of interest are labeled. WB, Western blot; αL1, antibody against ribosomal protein uL1.

A first step towards a specific readout for subunit assembly has been described recently. Two reporter strains have been generated possessing one r-protein of the large and the small subunit fused with green or red fluorescent proteins, respectively. While the reporter strain termed MCrg [41] harbors ribosomes with two labeled late assembly proteins (Supplementary Figure S1), in a second reporter strain MCrg* [42], two early assembly proteins were labeled (Supplementary Figure S2). Consequently, in MCrg, only fully assembled subunits are fluorescently marked, which allows for using that strain for identification of assembly inhibitors in small molecule screenings by comparing the fluorescence ratio (green/red) of untreated with treated reporter cells. In contrast, in MCrg*, both fully assembled and incomplete subunits are fluorescently marked, allowing for monitoring of assembly landscapes when ribosome profiles are analyzed fluorometrically [42]. Given the necessity to not only detect but also quantify subunit specific assembly defects, two new strains were created, which are the logical consequence of the two predecessors MCrg and MCrg*. The new strains possess either large (MCrgL) or small subunits (MCrgS) labeled with fluorescent proteins. In both strains, one early assembly protein (uL1 or uS15) is labeled with mCherry and one late assembly protein (bL19 or uS2) with mAzami. Both phenotypic and biochemical characterization of MCrgL and MCrgS, respectively, revealed that the strains show no defects at 37 or 42 °C and only a mild growth defect at very low temperature. In summary, we conclude that their growth properties and translation apparatuses are wild type-like (Figure 5).

**Figure 6:**
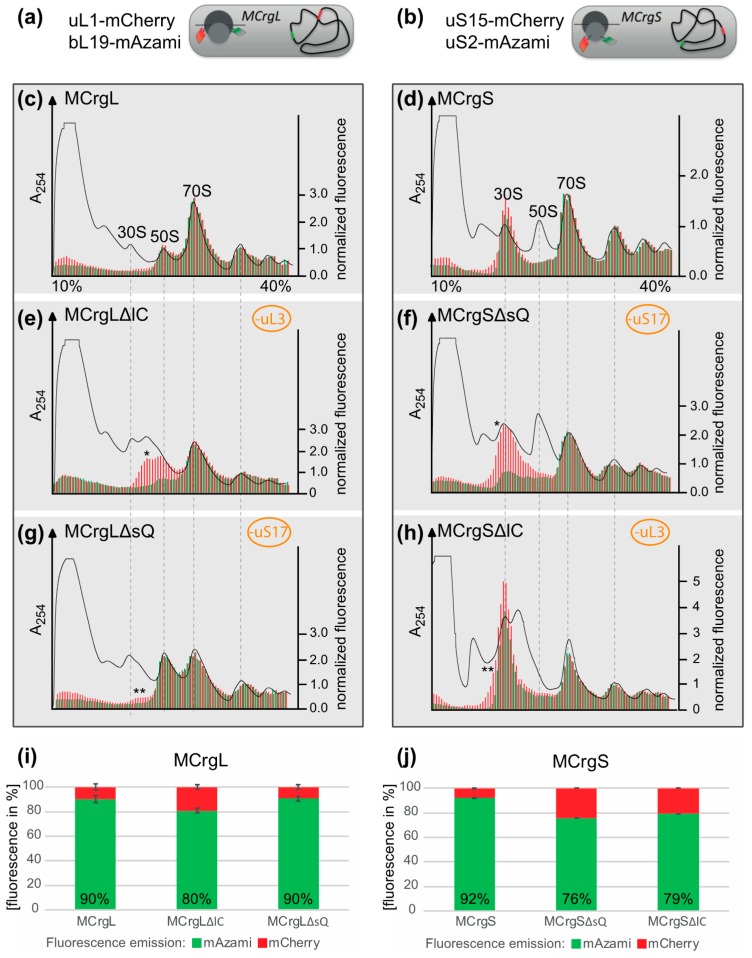
Ribosome profiles after sucrose density gradient centrifugation and fluorescence detection. (**a**,**b**) schematic drawing of MCrgL and MCrgS*, respectively. Depicted are the positions of the fluorescent proteins and their coding sequences. **Dark gray**: large ribosomal subunit; **light gray**: small ribosomal subunit; **green** barrel: EGFP; red barrel: mCherry; **gray** line: mRNA; curved **black** line: bacterial chromosome with red and green strips symbolizing coding sequences of mCherry and mAzami, respectively; (**c**–**h**) Ribosome profiles derived from MCrgL (**c**), MCrgLΔlC (**d**), MCrgLΔsQ (**e**), MCrgS (**f**), MCrgSΔsQ (**g**) and MCrgSΔlC (**h**); A_254_ profiles are given in black lines, mCherry and mAzami specific fluorescence intensities are given as red or green bars, respectively. Fluorescence intensities were normalized to the first polysome peak. Red and green fluorescence intensities were calculated and compared to each other, (**i**,**j**). Total mCherry and mAzami specific fluorescence emission of each strain’s profile is given in bar charts. mCherry signals were set to 100% and mAzami signals are given as relative values. Error bars show S.D. of two independent experiments. One asterisk indicates additional peak or shoulder within the subunit with the primary defect, two asterisks indicate additional peak or shoulder within the subunit was not targeted for assembly defect.

To test their capability to analyze subunit specific assembly defects, in both strains, conditional knock outs of ribosomal protein genes were introduced. The absence of the L-protein uL3 (encoded by *rplC*) or the S-protein uS17 (encoded by *rpsQ*) would render either large or small subunit assembly defective, as described previously [41,43,44]. While in MCrgL large subunit, mono- and polysomes contain red and green fluorescence (Figure 6c), in MCrgS small subunit, mono- and polysomes show red and green fluorescence (Figure 6d). In MCrgL, the absence of uL3 causes defects in the large subunit as becomes evident by a decrease of the green signal and an additional red fluorescence peak (Figure 6e). Defective small subunit assembly, in the absence of uS17, cannot directly be detected fluorometrically. However, directly caused defects in small subunit assembly seem to result in slight but detectable assembly defects of the large subunit as indicated by asterisks (Figure 6g). MCrgS, on the other hand, allows detection of small subunit assembly defects, which were caused by depletion of uS17, directly (Figure 6f). While the green fluorescence signal within the 30S region, which reflects the intact portion of the subunit, is decreased, the red fluorescence signal is broader and of higher intensity, clearly indicating a small subunit assembly defect. In the absence of uL3 (Figure 6h), defective large subunit assembly cannot be detected fluorometrically. However, again the subunit not affected by gene depletion, in this case the small subunit, shows a decrease in green fluorescence and a broadened red fluorescence peak with a left-sided shoulder. Quantitation of the fluorescence profiles confirms the observed tendencies (Figure 6i–j).

Taken together, fluorescence analyses of sucrose density gradient fractions derived from MCrgL and MCrgS enable detection and quantitation of assembly defects within the large or small subunit, respectively.

## 5. Materials and Methods

### 5.1. Media, Buffers, Antibodies and Antibiotics

LB-Medium (0.5% (*w*/*v*) yeast extract, 1% (*w*/*v*) tryptone, 86 mM NaCl; for growth on solid media additionally 1.5% (*w*/*v*) bacto agar); 5× M9 salts (63 mM Na_2_HPO_4_·7H_2_O, 110 mM KH_2_PO_4_, 43 mM NaCl, 94 mM NH_4_Cl); M9 medium (1× M9 salts, 2 mM MgSO_4_, 0.1 mM CaCl_2_, 0.4% (*w*/*v*) glucose); PBS (137 mM NaCl, 2.7 mM KCl, 10 mM Na_2_HPO_4_, 1.8 mM KH_2_PO_4_, pH 7.4). Horseradish peroxidase (HRP)-conjugated rabbit anti-sheep (CodeNo: 313-035-003; LotNo: 106383) and donkey anti-rabbit (CodeNo: 711-035-152; LotNo: 103871) and donkey anti-rabbit (CodeNo: 711-035-152; LotNo: 103871) secondary antibodies were from Jackson ImmunoResearch (West Grove, Pennsylvania, USA). HRP substrate for detection: 1 mL solution A + 100 µl solution B + 1 µL solution C (solution A: 0.1 mM TRIS (pH 8.6), 25 mg Luminol, 100 mL distilled H_2_O; solution B: 11 mg p-hydroxycoumaric acid in 10 mL DMSO; solution C: 30% H_2_O_2_ (*w*/*v*)). Antibiotics were used in concentrations as indicated: Ampicillin 100 µg/mL (Applichem-A0839, Darmstadt, Germany), kanamycin 50 µg/mL (Carl Roth-T832.4, Karlsruhe, Germany) and chloramphenicol 7 µg/mL (Sigma-C0378, St. Louis, MO, USA).

### 5.2. Plasmids and Bacterial Strains

*rpsQ* and *rplC* were amplified from genomic *E. coli* DNA, brought into DH5α-Z1 and isolated as described earlier [41]. 

MC4100 (F-[araD139]B/r Δ(argF-lac)169 lambda-e14-flhD5301 Δ(fruK-yeiR)725 (fruA25) relA1 rpsL150(strR) rbsR22 Δ(fimB-fimE)632(::IS1) deoC1); DY330 (W3110 Δ lacU169 gal490 λcI857 Δ (cro-bioA)) [45], DH5α-Z1 (F endA1 hsdR17(rk mk+) supE44 thi-1 recA1 gyrA relA1 Δ (lacZYAargF) U169 deoR Ф80 lacZΔ M15 LacR TetR and Spr).

### 5.3. λ-Red Recombineering

Fusion proteins of the ribosomal proteins with the FPs mAzami green and mCherry were generated by λ red recombineering and brought into strains of interest using P1-phage transduction as described previously. Genomic integration was confirmed by colony-PCR and DNA-sequencing. Gene deletions of *rpsQ* and *rplC* were also accomplished as described earlier [41].

### 5.4. Cell Growth Analyses

For growth on solid media, *E. coli* cells were grown at 37 °C until stationary phase in LB medium and diluted to a cell density of OD_600_ = 0.025. Serial dilutions with a dilution factor of 0.2 were prepared and transferred onto LB agar plates using a plating stamp. Plates were incubated at 20 °C, 30 °C, 37 °C and 42 °C until single colonies were visible. 

For growth in liquid media, stationary *E. coli* cells were grown until stationary phase and diluted to an initial OD_600_ = 0.025 for incubation at 42 °C and to OD_600_ = 0.05 for incubation at 37 °C and 20 °C. They were incubated in baffled flasks in a water bath incubator with a shaking frequency of 200 rpm until stationary or exponential phase was reached. Cell density was measured using a photometer (Ultrospec 3000, GE Healthcare, Little Chalfont, UK) and growth rates were calculated for periods of exponential growth. Growth analyses in liquid media were conducted in biological triplicates. 

### 5.5. Purification of Ribosomes by Sucrose Cushion Centrifugation

*E. coli* cells were cultured at 37 °C in LB medium until stationary phase was reached. Pre-cultures were diluted to an OD_600_ = 0.5 and incubated at 37 °C until an OD_600_ = 0.8. 250 µg/mL chloramphenicol was added to stop translation 5 min before harvesting. For this purpose, cultures were incubated on ice for 10 min and sedimented for 10 min at 4400 rpm and 4 °C. Pellets were resuspended in lysis buffer I (100 mM TRIS, 10 mM MgCl_2_, 100 mM NaCl, 15% (*w*/*v*) sucrose, 100 µg/mL chloramphenicol, pH 7.5), snap frozen in liquid nitrogen and stored at −80 °C. For further preparation, the frozen cell pellets were thawed on ice and resuspended in 3× volumes of lysis buffer II (10 mM MgCl_2_, 100 mM NaCl). Cells were then lysed using FastPrep^®^-24 (MP Biomedicals, Eschwede, Germany). Protein concentrations of the cleared lysates were determined by Bradford assay. In addition, 300 µL of cleared lysates were loaded on 700 µL of 20% sucrose cushion (20 mM TRIS, 10 mM MgCl_2_, 100 mM KCl, 5 mM β-mercaptoethanol, 20% sucrose (*w*/*v*), pH 7.5) and sedimented for 1 h 20 min at 65,000 rpm at 4 °C in an S140-AT rotor (Thermofischer Scientific, Waltham, MA, USA). Pellets harboring the ribosomes were resuspended in buffer III (10 mM TRIS, 12 mM MgCl_2_, 30 mM NaCl, 4 mM β-mercaptoethanol, pH 7.5). A_260_ values were determined using a spectrophotometer (NanoVueTM Plus UV/Visible Spectrophotometer (GE Healthcare).

### 5.6. Sucrose Gradient Centrifugation

*E. coli* cells of the different strains were cultured at 37 °C until stationary phase. The pre-cultures were washed 3 times and diluted in M9 minimal medium to OD_600_ = 0.05 and cultured to OD_600_ of about 0.3. Five minutes before harvesting, chloramphenicol (250 µg/mL) was added. The pellets were sedimented, snap frozen in liquid nitrogen and stored at −80 °C. For further preparation, pellets were resuspended in lysis buffer (10 mM TRIS, 10 mM MgCl2, 100 mM NH_4_Cl, 250 µg/mL chloramphenicol, 0.5 mM DTT, 1 mM PMSF, 1x TM CompleteTM (05056489001, (Roche-05056489001, Basel, Swiss) pH7.5) and lyzed using FastPrep^®^-24. A260 values of the cleared lysates were determined, the concentrations were adjusted to A_260_ = 20. In addition, 100 μL were then loaded on 10%–40% sucrose gradients and centrifuged at 4 °C and 41,000 rpm for 2 h 40 min using a Sorvall TH-641 rotor (Thermofischer Scientific, Waltham, MA, USA).

### 5.7. Polysome Analysis and Fluorometric Analysis of the Sucrose Fractions

Separated ribosomal populations were analyzed using a Teledyne Isco gradient reader (Teledyne ISCOr, Lincoln, Nebraska, USA). A254 profiles were recorded and the obtained fractions were collected in 96-well plates (5 drops per well) for the following fluorometric analyses. mAzami and mCherry specific fluorescence was determined using Infinite F500 (Tecan, Männedorf, Swiss) fluorescence microplate reader (filter combinations 485/535 nm and 535/612 nm, respectively). The fluorescence intensities were normalized to the first polysome peak.

## 6. Conclusions

The intricate assembly of the macromolecular complex “ribosome” offers many distinct interference points. Even though the structure of ribosomes is highly conserved across domains, a number of specific features of the biogenesis of the bacterial ribosomes exist. Only recently suitable methods have been developed to trace assembly inhibitors. We can expect that the near future will reveal inhibitors specifically blocking ribosome assembly in bacteria, which might pave the way for development of urgently needed new therapeutics against bacterial infections.

## Supplementary Materials

The following are available online at www.mdpi.com/2079-6382/5/2/18/s1. Figure S1: Ribosome profile analysis using MCrg, Figure S2: Ribosome profile analysis using MCrg*.

## Abbreviations

The following abbreviations are used in this manuscript:

Da	molecular mass in Dalton
EGFP	Enhanced green fluorescent protein
LB	Lysogeny broth (medium)
LB agar	LB and agar containing breeding grounds
rRNA	ribosomal RNA
PTC	peptidyl transferase center
tRNA	transfer RNA
TRIS	Tris(hydroxymethyl)-aminomethan
aa-tRNA	aminoacyl tRNA
GTP	guanosin triphosphate
GDP	guanosin diphosphate
EF-Tu	elongation factor thermo-unstable
EF-G	elongation factor G
FA	fusidic acid
mAzami	Monomeric green fluorescent protein
mCherry	Monomeric red fluorescent protein
OD_600_	Optical density at 600 nm wavelength
S.D.	standard deviation
SDS PAGE	Sodium dodecyl sulfate poly acrylamide gel electrophoresis
