# Supplementary material for: Ribosome Assembly as Antimicrobial Target
Source: Antibiotics (Basel). 2016 May 27;5(2):18. doi: 10.3390/antibiotics5020018 (PMC4929433; doi:10.3390/antibiotics5020018)
Supplement: Supplementary file 1 [file antibiotics-05-00018-s001.pdf]

Supplementary Materials: Ribosome Assembly as Antimicrobial Target

Rainer Nikolay, Sabine Schmidt, Renate Schlömer, Elke Deuerling and Knud H. Nierhaus

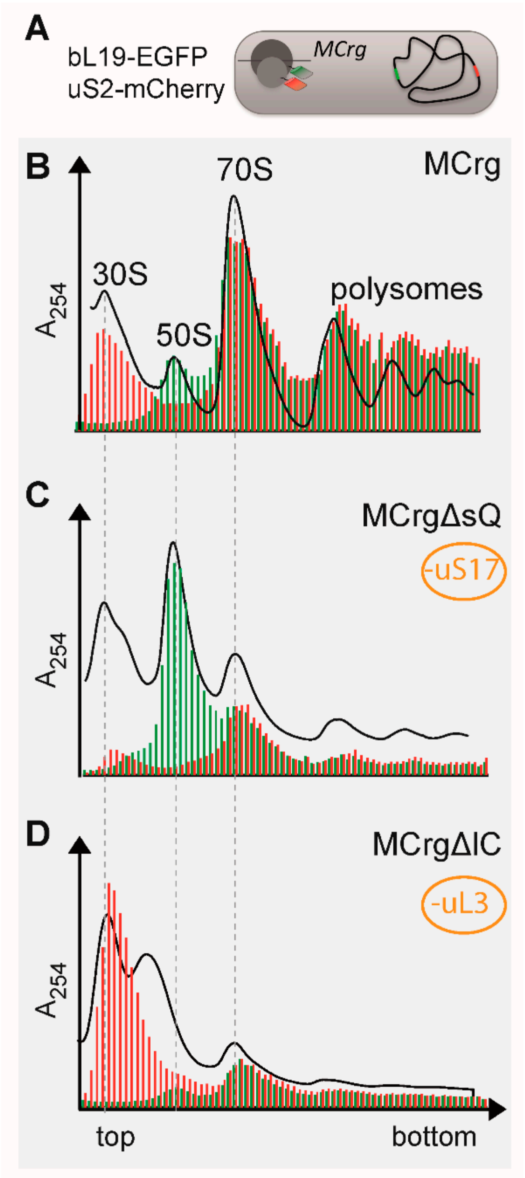

Figure S1. Ribosome profile analysis using MCrg (**a**) Schematic drawing of MCrg illustrating where the fluorescent proteins and their coding sequences are positioned. **Dark gray**: large ribosomal subunit; light gray: small ribosomal subunit; green barrel: EGFP; **red** barrel: mCherry; **gray** line: mRNA; curved **black** line: bacterial chromosome with **red** and **green** strips symbolizing coding sequences of mCherry and EGFP, respectively; Polysome profiles derived from (**b**) MCrg; (**c**) MCrgΔsQ and (**D**) MCrgΔlC. A254 profiles are given in black lines, mCherry and mAzami specific fluorescence intensities are given as red or green bars, respectively. Fluorescence intensities were normalized to the first polysome peak. Adopted from Nikolay *et al.*, 2014 [1].

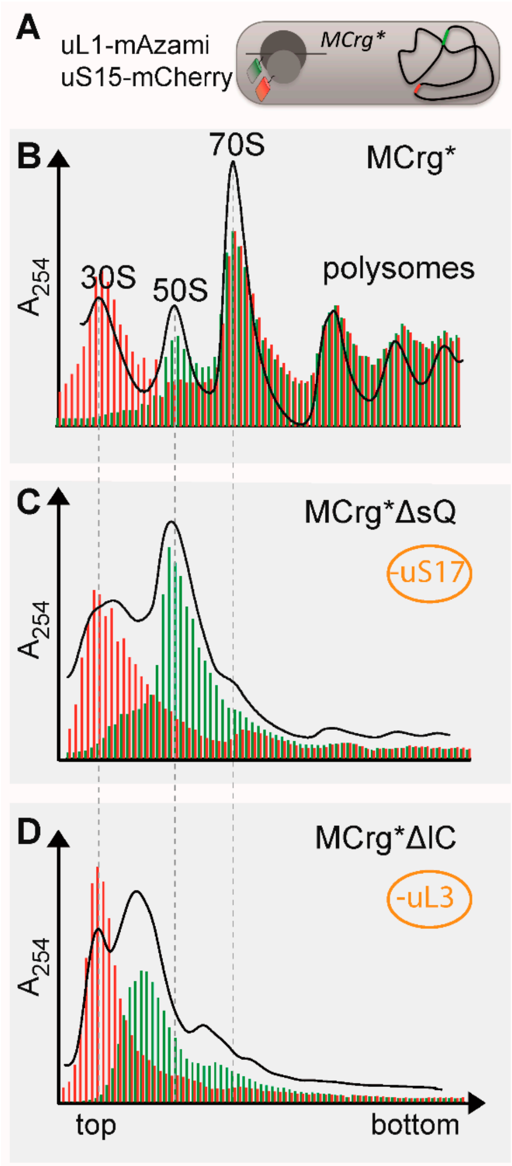

Figure S2. Ribosome profile analysis using MCrg*. (**a**) Schematic drawing of MCrg* illustrating where the fluorescent proteins and their coding sequences are positioned. **Dark gray**: large ribosomal subunit; light gray: small ribosomal subunit; **green** barrel: mAzami; **red** barrel: mCherry; **gray** line: mRNA; curved black line: bacterial chromosome with **red** and **green** strips symbolizing coding sequences of mCherry and mAzami, respectively; Polysome profiles derived from (**b**) MCrg*; (**c**) MCrg*ΔsQ and (**D**) MCrg*ΔlC. A254 profiles are given in black lines, mCherry and mAzami specific fluorescence intensities are given as red or green bars, respectively. Fluorescence intensities were normalized to the first polysome peak. Adopted from Nikolay *et al.*, 2015 [2].

References

1. Nikolay, R.; Schloemer, R.; Schmidt, S.; Mueller, S.; Heubach, A.; Deuerling, E. Validation of a fluorescence-based screening concept to identify ribosome assembly defects in escherichia coli. *Nucleic acids research* **2014**, *42*, e100, doi:10.1093/nar/gku381.
2. Nikolay, R.; Schloemer, R.; Mueller, S.; Deuerling, E. Fluorescence-based monitoring of ribosome assembly landscapes. *BMC Mol. Biol.* **2015**, *16*, 3, doi:10.1186/s12867-015-0031-y.
